# Periosteal reaction: a natural extracellular matrix barrier to prevent cancer-bone invasion

**DOI:** 10.1093/procel/pwaf050

**Published:** 2025-06-18

**Authors:** Zhuo Chen, He-Jing Zhang, Zhengrui Chang, Yi Tang, Stephen J Weiss, Lingxin Zhu

**Affiliations:** State Key Laboratory of Oral & Maxillofacial Reconstruction and Regeneration, Key Laboratory of Oral Biomedicine Ministry of Education, Hubei Key Laboratory of Stomatology, School & Hospital of Stomatology, Wuhan University, Wuhan 430079, China; State Key Laboratory of Oral & Maxillofacial Reconstruction and Regeneration, Key Laboratory of Oral Biomedicine Ministry of Education, Hubei Key Laboratory of Stomatology, School & Hospital of Stomatology, Wuhan University, Wuhan 430079, China; State Key Laboratory of Oral & Maxillofacial Reconstruction and Regeneration, Key Laboratory of Oral Biomedicine Ministry of Education, Hubei Key Laboratory of Stomatology, School & Hospital of Stomatology, Wuhan University, Wuhan 430079, China; Division of Genetic Medicine, Department of Internal Medicine, University of Michigan, Ann Arbor, MI 48109, USA; Life Sciences Institute, University of Michigan, Ann Arbor, MI 48109, United States; Division of Genetic Medicine, Department of Internal Medicine, University of Michigan, Ann Arbor, MI 48109, USA; Life Sciences Institute, University of Michigan, Ann Arbor, MI 48109, United States; State Key Laboratory of Oral & Maxillofacial Reconstruction and Regeneration, Key Laboratory of Oral Biomedicine Ministry of Education, Hubei Key Laboratory of Stomatology, School & Hospital of Stomatology, Wuhan University, Wuhan 430079, China

The periosteum generally refers to the outer layer of bone, a specialized tissue primarily composed of fibroblasts and concentrically arranged type I collagen fibers that also serves as a reservoir for periosteal stem cells (Debnath et al., 2018). During physiological bone formation, periosteal stem cells contribute to osteogenesis via the intramembranous pathway, involving cell proliferation and osteogenic differentiation. Following bone damage, however, periosteal stem cells first differentiate into chondrocytes to generate a cartilaginous matrix prior to osteogenic commitment where they participate in bone repair through a process termed, endochondral ossification (Jeffery et al., 2022). Either of these processes lead to reactive expansion of the periosteum and new bone formation, a phenomenon known as the “periosteal reaction” (Rana et al., 2009). Since its discovery in 1739, the periosteal reaction has been widely reported for its positive role in bone formation, fracture repair, and bone regeneration (Allen et al., 2004; Rana et al., 2009). Additionally, periosteal reaction is a typical manifestation in bone tumors such as osteosarcoma (Murphey et al., 2004) and is closely associated with the formation of osteophytes in osteoarthritis (Roelofs et al., 2020). However, existing studies primarily focus on periosteal reaction during bone injury, while potential interactions between bone-invasive cancer cells and the periosteum during tumor progression remain unknown.

To address this gap in knowledge, Nakamura et al. evaluated patients with head and neck squamous cell carcinoma (HNSCC) (Nakamura et al., 2024). They found that periosteal thickness significantly increased near tumors that failed to invade bone, whereas the periosteum was completely lost under conditions where tumors successfully infiltrated bony tissues. These results led the investigators to propose a potential role for the periosteal reaction in regulating cancer-related bone invasion. The authors employed HNSCC mouse models with intact or mechanically scratched periosteum to respectively simulate the pre-invasion and invasion stages of tumor progression. As opposed to cancer cells approaching an intact periosteum, bone invasion and destruction was exacerbated when the periosteum had been previously damaged. When cancer cells faced an intact periosteum, the number of periosteal cells significantly increased, accompanied by the upregulation of gene expression, suggesting that periosteal cells are highly responsive in the tumor invasion microenvironment. To implicate periosteal stem cells more specifically in these responses, the authors took advantage of recent studies demonstrating that these stem cells express cathepsin K (Ctsk), a cysteine proteinase long thought to play a dominant role in osteoclast-mediated bone resorption (Chai et al., 2022). As such, they administered diphtheria toxin mice engineered to express a diphtheria toxin receptor in *Ctsk-Cre*-positive cells (i.e., *Ctsk-Cre*/*R26-*iD*TR* mice) in tandem with wild-type bone marrow cell transplantation to obviate any effect on osteoclast function. Under these conditions, the periosteum was unable to protect bone from invading cancer cells, supporting the contention that periosteal stem cells play a dominant role in protecting bone from HNSCC invasion and destruction. These findings collectively indicate that periosteal stem cells play a required role in the periosteal reaction and in the consequent resistance to tumor-induced bone invasion.

Finally, the authors sought to identify the potential mechanisms underlying the protective effects exerted by the periosteum. Examining the transcriptional changes taking place as HNSCC cells approached the periosteum, they identified tissue inhibitor of metalloproteinases-1 (TIMP1) as a significantly upregulated gene expressed in periosteal cells during the pre-invasive stage of HNSCC progression. Traditionally, TIMP1 (as well as the other closely related members of this gene family, i.e., TIMP2, 3 and 4) inhibit the activity of matrix metalloproteinases (MMPs) through non-covalent binding to their active sites (Jackson et al., 2017). In turn, MMPs play critical roles in tissue remodeling and tumor bone invasion as a function of their ability to collectively degrade virtually all extracellular matrix (ECM) components (Jackson et al., 2017). With specific regard to TIMP1, it can inhibit all secreted MMPs as well as a subset of the membrane-anchored MMPs, i.e., MT4-MMP and MT6-MMP (Jackson et al., 2017), thereby exerting a suppressive effect on ECM degradation and tumor progression. Further studies revealed that TIMP1 knockout mice displayed a normal periosteum at steady state, but were unable to thicken the tissue when confronted with HNSCC tumor cells. Under these conditions, the TIMP1 knockout periosteum also lost type I collagen, its dominant ECM component, while likewise presenting with an increased susceptibility to tumor bone invasion. Though global knockout mice are likely to have other defects unrelated to periosteal function, remarkably, administering recombinant TIMP1 rescued the bone-protective phenotype. These results are most consistent with a model wherein TIMP1 not only prevents HNSCC-triggered, MMP-dependent type I collagen degradation in the periosteum, but also supports periosteal thickening that presumably occurs in response to increased collagen deposition, thereby blocking cancer invasion into the surrounding bone.

In considering the factors responsible for increasing TIMP1 levels in the periosteum, previous work demonstrated that under hypoxic conditions, hypoxia-inducible factor 1 alpha (HIF1α) can directly promote TIMP1 expression (Dery et al., 2023). The authors then confirmed elevated expression of HIF1α in the pre-invasive stage of HNSCC, along with its direct interaction with TIMP1. In tandem with these observations, they reported a strong correlation between HIFα and TIMP1 co-expression *in vivo* as well as in human HNSCC lesions.

As illustrated in Fig. 1, this study proposes that tumor-derived HIF1α induces periosteal TIMP1, forming a protective barrier that inhibits bone invasion. Like all tantalizing observations, these striking and important results beg a number of outstanding questions. How do encroaching, pre-invasive HNSCCs trigger periosteal thickening at a distance? How do invasive HNSCCs eventually circumvent the protective effects provided by periosteal thickening? Further, while the *Ctsk-Cre*-driven elimination of periosteal stem cells and their progeny support a key role for these cells *in vivo*, recent studies indicate that *Ctsk-Cre* is more widely expressed than previously reported, including osteocytes, blood vessels and tenocytes (Chai et al., 2022) and may preferentially target periosteal progenitors rather than stem cells themselves (Perrin et al., 2024). Therefore, although the authors have ruled out an effect on Ctsk^+^ osteoclasts, potential off-target effects of *Ctsk-Cre* on other cell populations cannot be ruled out. Likewise, while studies using TIMP1 knockout mice and reconstitution are intriguing, the actual TIMP1 target(s) and their tissue of origin remain to be identified.

**Figure 1.**
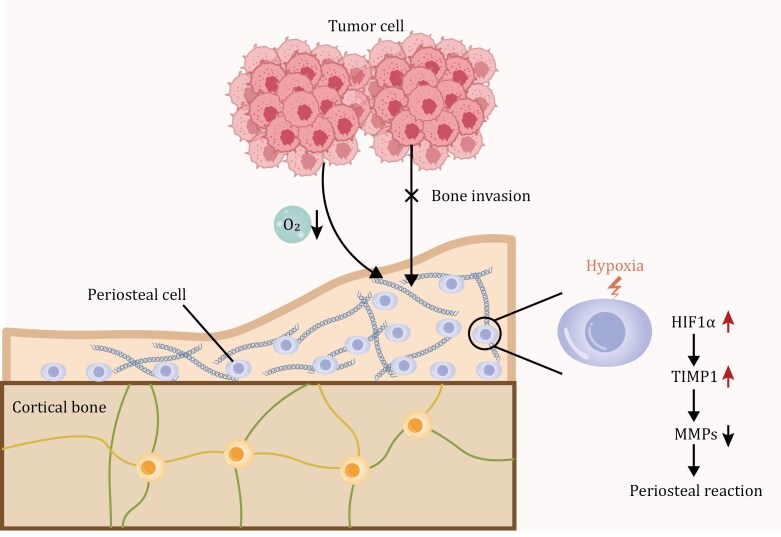
**The interaction mode between cancer and periosteal reaction during tumor bone invasion.** The elevated levels of HIF1α in the tumor hypoxic microenvironment lead to elevated production of TIMP1 by periosteal cells. The periosteal TIMP1 subsequently inhibits the activity of MMPs, limits periosteal extracellular matrix degradation and enhances tumor-induced periosteal reaction, which essentially impedes the occurrence of tumor bone invasion.

Nevertheless, the protective role played by TIMP1 in preventing periosteal ECM remodeling axis during HNSCC bone invasion provides new directions for future research. As the authors have noted, the role of TIMP1 in HNSCC suggests that it may serve as a key therapeutic target for inhibiting bone invasion in HNSCC. However, caution need be exercised considering TIMP1 as a therapeutic intervention. As the authors correctly pointed out, TIMP1 has been shown to promote the formation of premetastatic niches in other cancer models (Grünwald et al., 2016; Seubert et al., 2015). TIMP1 is also capable of exerting more complex effects *in vivo* through MMP-independent pathways (Coates-Park et al., 2024). In this regard, TIMP1 has been reported to promote tumor cell growth and inhibit apoptosis through MMP-independent mechanisms in various cancer types, thereby facilitating tumor proliferation and migration (Coates-Park et al., 2024; Priego et al., 2025). As the authors pointed out, experiments using TIMP1 mutants that are devoid of MMP inhibitory activity, but retain the ability to affect multiple cell functions, ranging from blocking apoptotic processes to triggering Wnt signaling, are particularly important (Egea et al., 2012; Nalluri et al., 2015). Alternatively, even if TIMP1 is functioning as a proteinase inhibitor, the identity of the target MMPs responsible for periosteal matrix remodeling remain to be determined. While several of the MMPs identified in their report have type I collagenolytic potential, it should be noted that mice do not express MMP1, one of the dominant TIMP1-sensititve collagenases found in humans (Zhang et al., 2016). Similarly, determining whether TIMP1 is targeting MMPs arising in the periosteum itself, infiltrating myeloid cells, bone-resorbing osteoclasts or the carcinoma cells themselves will be informative to providing further mechanistic insight. Moreover, the study by Nakamura et al. focused primarily on the role of periosteal reaction in bone invasion by HNSCC. Whether this ECM barrier also plays a role in local bone invasion by other types of tumors remains unknown. In fact, other tumor types—such as basal cell carcinoma—also tend to invade local bone tissue (Russell et al., 2022). However, most bone metastases arise by hematogenous spread (Zhang et al., 2024), and Nakamura et al. noted that TIMP1 did not exert protective effects in this scenario. While more work will be needed to address outstanding concerns, the important studies carried out by Nakamura et al. shed new light on a heretofore-overlooked HNSCC-periosteal axis.

## Data Availability

Not applicable.

## Abbreviations

Ctsk: cathepsin K
ECM: extracellular matrix;
HIF1α: hypoxia-inducible factor 1 alpha
HNSCC: head and neck squamous cell carcinoma
MMP: matrix metalloproteinase;
TIMP1: tissue inhibitor of metalloproteinases-1
